# *Bacillus velezensis* B105-8, a potential and efficient biocontrol agent in control of maize stalk rot caused by *Fusarium graminearum*

**DOI:** 10.3389/fmicb.2024.1462992

**Published:** 2024-10-16

**Authors:** Shuang Wang, Pinjiao Jin, Yanyan Zheng, Wang Kangkang, Jiaxin Chen, Jiansheng Liu, Yonggang Li

**Affiliations:** ^1^Heilongjiang Academy of Black Soil Conservation and Utilization/Heilongjiang Black Soil Conservation Engineering and Technology Research Center, Harbin, China; ^2^State Key Laboratory of Desert and Oasis Ecology, Key Laboratory of Ecological Safety and Sustainable Development in Arid Lands Xinjiang Institute of Ecology and Geography, Chinese Academy of Sciences, Urumqi, China; ^3^College of Plant Protection, Northeast Agricultural University, Harbin, China; ^4^Heilongjiang Guohong Energy Conservation and Environmental Protection Co., Ltd., Harbin, China

**Keywords:** maize stalk rot, *Bacillus velezensis*, antifungal activities, biological control, active substances

## Abstract

**Introduction:**

Maize stalk rot (MSR), caused by *Fusarium graminearum*, is the most serious soil borne disease in maize production, seriously affecting maize yield and quality worldwide. Microbial biocontrol agents are the best means of controlling MSR and reducing the use of chemical fungicides, such as *Bacillus* spp.

**Methods and results:**

In the study, a soil-isolated strain B105-8 was identified as *B. velezensis* (accession No. PP325775.1 and No. PP869695.1), demonstrated a broad spectrum against various pathogens causing maize diseases, which effectively controlled MSR, exhibited a high control efficacy of more than 60% and growth-promoting effect in the pot plant. B105-8 could effectively improve soil urease (S-UE), invertase (S-SC), and catalase (S-CAT) activities. S-NP activity showed an initial increase with a peak of 20,337 nmol/h/g, followed by a decrease, but activity remained significantly better than control treatment with chemical fungicides. The application of B105-8 repaired the damage caused by *F. graminearum* on soil activity. The antifungal compound B-1, extracted from B105-8, was purified using a protein purifier, revealing inhibitory effects against *F. graminearum*. Mass spectrometry analysis indicated the potential presence of C14 Bacillomycin, C15 Iturin, C15 Mycosubtilin, C17, and C15 fengycin in B-1. In pot experiments, a 5 μL/mL concentration of B-1 exhibited 69% control on MSR, enhancing maize root elongation, elevation, and fresh weight. At 10 μL/mL, B-1 showed 89.0 and 82.1% inhibition on spore production and mycelial growth, causing hyphal deformities.

**Discussion:**

This study presents the innovative use of *B. velezensis*, isolated from maize rhizosphere in cold conditions to effectively control MSR caused by *F. graminearum*. The findings highlight the remarkable regional and adaptive characteristics of this strain, making it an excellent candidate to fight MSR in diverse environments. In conclusion, *B. velezensis* B105-8 demonstrated potential as a biocontrol agent for MSR.

## 1 Introduction

Maize (*Zea mays* L.), recognized as a globally significant cereals, serves as a vital source of feed/fodder for livestock (Karuppiah et al., 2022). Maize stalk rot (MSR) is one of the most widespread and destructive soil-borne diseases in maize, resulting in significant yield reductions, diminished grain quality, and difficulties in post-harvesting (Wang et al., 2022; Cheng et al., 2019). MSR is caused by multiple pathogens, including *Fusarium graminearum* (Chen et al., 2019), *F. thapsinum* (Zhang et al., 2021a)*, F. kyushuense* (Cao et al., 2021a), *F. culmorum* (Xia et al., 2022), *F. brachygibbosum*in (Shan et al., 2017), *F. nelsonii* (Zhang et al., 2021b), *Epicoccum latusicollum* (Xu et al., 2022), *F. cerealis* (Shan et al., 2018), in China, *Phaeocytostroma ambiguum* in Brazil (Aguiar et al., 2016), *F. falciforme* in Mexico (Douriet-Angulo et al., 2019). Among them, *F. graminearum* is the most predominant causative agent of MSR (Chen et al., 2019; Karuppiah et al., 2022), which tends to be more common in higher-yielding hybrids that produce large ears (Zhang et al., 2016). *F. graminearum* also releases mycotoxins, such as deoxynivalenol and zearalenone, which pose hazards to the health of both humans and livestock (Mohammadi et al., 2011). Moreover, *F. graminearum* can survive in the soil for numerous years, rendering it challenging to control. In China, losses associated with MSR span from 5 to 80%, thus, control of toxigenic *F. graminearum* is challenging (Yu et al., 2017).

Traditionally, the management of MSR has been reliant on the utilization of resistant cultivars and application of chemical fungicides (Shin et al., 2014). Nevertheless, current agricultural techniques exhibit limitations in their efficacy. Resistance amongst maize cultivars to stalk rot remains inadequate, and the process of cultivating resistant maize varieties is remarkably time-consuming (Chen et al., 2019). As the pathogens spread and invade plants via the soil matrix, the application of fungicides proves ineffective in managing MSR, and may pose hazards to humans and the environment (van der Werf, 1996; Wang et al., 2022).

Biocontrol strategies represent optimal and environmentally responsible strategies for disease control and maize yield enhancement (Cao et al., 2021b; Karuppiah et al., 2022) and have attracted attention as an eco-friendly method for controlling MSR using beneficial microorganisms, including *Bacillus methylotrophicus* (Chen et al., 2019), *Trichoderma asperellum* (Lu et al., 2020)*, B. cereus* (Lizárraga-Sánchez et al., 2015). *Bacillus* spp. can form endospores and resist hostile conditions, such as desiccation, heat, and ultraviolet irradiation. Thus, the genus Bacillus-based products predominate over other biocontrol microorganisms and represent the best candidates for the development of efficient biopesticides (Torres et al., 2017). There have been >100 commercial products of Bacillus formulations registered in China so far. Among *Bacillus* species, *Bacillus* spp. are widespread in natural habitats including soils and plants (Cao et al., 2021b). Potted experiments showed that *B. velezensis* (RC116) isolated from tomato rhizosphere soil in Guangdong Province, China, had an 81% biological control effect on tomato bacterial wilt, significantly promoting tomato plant growth (Dong et al., 2023). A suspension of soil isolated strain *B.velezensis* (BVE7) can effectively control soybean root rot (SRR), with a maximum control efficiency of 75.13%, reducing the damage caused by fungi and the severity of soybean root rot (Sun et al., 2023). *B. velezensis* provides bacterial resources and theoretical basis for biological control of soil borne diseases.

As biological control agents, *Bacillus* spp. produce active secondary metabolites, which may promote the healthy growth of plants via various mechanisms, such as direct antibiosis, the suppression of several soil-borne plant pathogens, plant growth promotion, and the induction of systemic resistance in plant hosts (Cao et al., 2021b; Goecks et al., 2013; Rasul et al., 2019). Moreover, some *Bacillus* spp. possess strong colonizing capabilities and can be easily handled. In addition, they fight pathogens for niche or nutritional requirements and directly generate active secondary metabolites against various plant pathogens, such as iturin, surfactin, and fengycin (Geissler et al., 2017; Fira et al., 2018). Furthermore, the production of antimicrobial peptides (AMPs) is a major defense mechanism against pathogen infestation (Ratzka et al., 2012). Thus, *Bacillus* spp. were screened as excellent biocontrol agents for MSR in the study.

Therefore, the objectives of this study were to (i) isolate and identify *Bacillus* spp. exhibiting antagonism against MSR; (ii) evaluate the antagonistic efficacy of the strain for controlling MSR *in vitro*; (iii) analyze the antifungal active components and clarifing the mechanism of inhibiting fungi.

## 2 Materials and methods

### 2.1 Isolation of bacterial strains

One hundred and twenty different bacterial strains were isolated from healthy maize plants (MSR disease resistant inbred line 18 N10118) in the rhizosphere soil of the corn field during V11 the maize rhizosphere soil of Harbin (126.93° E, 45.77° N), China, using the soil dilution method (Li et al., 2020b). A single bacterial colony was picked and purified after 72 h of incubation at 28°C by repeated streaking on beef extract peptone medium (BPM) (Beijing Aoboxing Biology Technology Co., Ltd.) plates.

### 2.2 Screening of biological control bacteria against *Fusarium graminearum* causing MSR

The target pathogen *F. graminearum* isolate FGM9 (accession no. MT563086) was isolated and preserved by our team and was the predominant population of MSR-causing pathogens in Northeast China, representing 23.2% of the total number of fungi isolated (Liu et al., 2023). A preliminary screening of antagonistic bacteria was conducted using the confrontation methodology (Li et al., 2020a). Following 48 h of culture of the tested bacterial strains on nutrient agar medium (NA), plates at 28°C and inoculated at a distance of 3 cm from the center of a potato dextrose agar (PDA) plate (diameter 9 cm), utilizing the parallel streak method. A 0.7-cm-diameter disk of *F. graminearum* was inoculated in the center of the PDA plate and cultivated at 26°C for 5 days with three replications. The maximum and minimum radii of *F. graminearum* colonies were calculated to determine the antagonistic activity of the tested bacterial strains (Wang et al., 2020a). Strains with a ratio of longest-to-shortest radius exceeding 1 were selected for a thorough re-screening process.

### 2.3 Re-screening of biocontrol bacteria

The most advantageous bacteria exhibiting antagonistic effects were initially screened in pots to determine the efficacy of biological control, which obtained the target biocontrol bacterial strain. The precise methodology was as follows: the bacteria were inoculated into NA sterilized liquid and shaken at 180 r/min for 48 h at 28°C. The resulting fermentation liquor was subsequently subjected to centrifugation (5,000 r/min, 4°C) for 30 min. The supernatant was discarded, and the remainder bacteria were washed three times with sterilized deionized water; then, the bacteria were adjusted to 10^8^ CFU/mL for subsequent use (Sun et al., 2018; Zhou et al., 2024). Inoculum of *F. graminearum* was prepared by inoculating sorghum seeds as described in our previous study (Li et al., 2018).

Through a potted experiment, bacterial strains exhibiting a maximum radius/minimum radius ratio of more than 1 have been selected for re-screening to evaluate their control efficacy. Two-thirds of the sterilized culture medium (peat: vermiculite = 2:1) was transferred to the cultivation bowl (10 cm × 10 cm). Using the sorghum grain root embedding method, 1 g of crushed sorghum grain powder with mycelium covered surface and added to 9 mL of sterile water. The mixture was shaken for 30 min at 28°C and 150 r/min, and gradually diluted to 10^−4^. Draw 100 μL of suspension and apply it to a PDA plate. Calculate the spore count for different gradient, and make the number of spores contained in each gram of expansion is approximately 3 × 10^5^ for subsequent inoculation (Chang et al., 2020; Zeng et al., 2024). The 1 g sorghum grains were covered with a layer of sterilized culture medium 0.5 cm in thickness and uniformly sown with 10 maize seeds soaked in a suspension of biocontrol bacteria (1 × 10^8^ CFU/mL) for 12 h. Subsequently, a 2 cm thick layer of medium was applied and the potted plant was transferred to a white porcelain dish. Tap water was subsequently added to the lower portion of the culture medium until saturation was achieved. On the 10th day, the root length, shoot length, and fresh weight of each plant were recorded. The incidence was calculated by surveying the number of diseased plants.

Disease reduction (%) was calculated as follows: (disease index of untreated control − disease index of the treatment) / (disease index of the untreated control) × 100.

### 2.4 Antifungal spectrum

Utilizing the described confrontation method, the antifungal spectrum of selected biocontrol bacterial strain B105-8 against eight species of pathogenic fungi was assessed and the specific operation method is as above. The diameter of target fungi inoculated with biocontrol strains was used as the treatment group, while the diameter of non biocontrol strains was used as the blank control.

The antagonistic effect of biocontrol bacterium B105-8 was evaluated against eight fungal pathogens causing maize disease, including *Bipolaris zeicola* (accession No. PQ289216), *Fusarium equiseti* (accession No. PQ289217), *F. graminearum* (accession No. PQ289218), *F. avenaceum* (accession No. PQ289219), *F. subglutinans* (accession No. PQ289220), *Nigrospora oryzae* (accession No. PQ289221), *F. verticillioide* (accession No. PQ289222), *Exserohilum turcicum* (accession No. PQ289223). The aforementioned pathogenic fungi were obstained by the plant pathology laboratory of Northeast Agricultural University, China. The measurement and evaluation methods were the same as those described in Section 2.2.

### 2.5 Identification of biocontrol bacteria B105-8

The biocontrol bacterium strain B105-8 exhibiting superior antagonistic effect was subjected to a detailed analysis of its morphological characteristics using nutrient agar (NA) in combination with a scanning electron microscope (SU8010, Hitachi, Tokyo, Japan). Detailed observations of the physiological and biochemical features of strain B105-8 were comprehensively undertaken in accordance with previously mentioned by Schaad et al. (2001), Buchanan (1984), and Dong and Cai (2001).

The following steps were meticulously executed to prepare a sample for analysis in a scanning electron microscope: the bacterium was activated on the BPM medium by the addition of glutaraldehyde for a fixation of 1.5 h. Subsequently, the treated sample was exposed to numerous rounds of rinsing with PBS to eliminate any unbound molecules, followed by dehydration using ethanol. Next, the sample was dried utilizing a freeze dryer (Hitachi, ES-2030). Finally, the sample was adhered to the sample holder of the scanning electron microscope using conductive tape, followed by the application of a metallic film on its surface for further inspection.

The physiological and biochemical traits were examined utilizing the following indicators: Gram staining, catalase test, hydrolysis of casein, starch, gelatin, and sulfur amino acids, nitrate reduction, the Voges-Proskauer (V-P) test, and Methyl red (MR) test, and the utilization of glucose, mannitol, fructose, sucrose, lactose, cellobiose, arabinose, xylose, maltose, rhamnose, citrate, sorbitol, inositol, and malonate using the physiological and biochemical traits kits (Guangdong Huankai Microbial Sci.&Tech. Co., Ltd., Guangdong, China).

The genomic DNA of strain B105-8 was extracted employing a plant genome DNA extraction kit (Beijing ComWin Biotech Co., Ltd.). The genomic DNA of the strain was extracted, and 16S ribosomal RNA gene and DNA gyrase subunit B (*gyrB*) gene were amplified and sequenced using the primers 1492R/27F (1492R: 5′-GGTTACCTTGTTACGACTT-3′; 27F: 5′-AGAGTTGATCCTGGCTCAG-3′) (Lane, 1991), and *gyrB-F*/*gyrB-R* (*gyrB-F*: 5′-MGGCGGYAAGTTCGATGACAAYTC-3′; *gyrB-R*: 5’-TRATBKCAGTCARACCTTC RCGSGC-3′)(Sara and David, 2004). Amplification experiments were conducted using a PCR mix kit (CW0556, Beijing ComWin Biotech Co., Ltd., Beijing) in a total reaction volume of 50 μL (Li et al., 2020b). The PCR program was implemented as follows: 5 min at 94°C; 36 cycles of 1 min at 94°C, 1 min at 58°C, and 1.5 min at 72°C; and a final extension step of 10 min at 72°C. The amplified product was sequenced by Genewiz Biotechnology Co., Ltd. (Suzhou, China) and analysed by BLAST (https://blast.ncbi.nlm.nih.gov/ Blast.cgi). Phylogenetic trees of representative isolates were constructed using PhyloSuite v1.2.2 and MrBayes methodology, as described by Zhang et al. (2020). The constructed trees were then edited in FigTree v1.4.4 (http://tree.bio.ed.ac.uk/software/figtree/).

### 2.6 Reduction MSR following application of B105-8

An experiment on the efficacy of potting was conducted in the greenhouse of the Northeast Agricultural University School of Agriculture. Mature and undamaged maize seeds were selected, exposed to 70% ethanol treatment for 3 min, and subsequently rinsed with sterile water and dried to prepare for utilization.

The preparation of the inoculum is as follows: the biocontrol bacteria B105-8 was cultured in liquid LB medium for 48 h and subsequently diluted to a concentration of 1 × 10^8^ CFU/mL (an OD 600 nm of 0.1) utilizing 0.9% normal saline solution as a diluent for storage. The inoculum of *F. graminearum* was prepared by inoculating sorghum seeds following the protocol described by Li et al. (2018).

The study comprised four treatments, each replicated thrice. CK treatment involved inoculating only with the pathogenic fungus; T1 referred to inoculation of the pathogenic fungus along with 1 mL/plant of biocontrol bacteria; T2 denoted to inoculation of the pathogenic fungus with 2 mL/plant of biocontrol bacteria; T3 indicated inoculation of the pathogenic fungus with 3 mL/plant of biocontrol bacteria.

The pots were placed in a greenhouse with a temperature of 23 ± 3°C. A total of fifty plants were inoculated and after inoculation, conventional methods were employed in managing the seeds (cv. Dongnong 264). The disease’s severity was assessed by evaluating the growth status of maize’s roots utilizing a scale ranging from 0 to 4: Where 0 = no symptoms; 1 = Root lesions account for less than 1/4 of the total, with white roots being the main cause; 2 = Root lesions account for less than 1/4–1/2 of the total, with white and brown areas equivalent; 3 = Root lesions account for less than 1/2–3/4 of the total, with white areas smaller than brown areas; and 4 = Root lesions account for more than 3/4 of the total, as the root decays in its entirety. The experiment was performed twice under identical experimental conditions. The disease index (DI) for MSR was calculated as follows:

DI = ∑(number of diseased plants at each level × number of relative ratings)/ (total number of surveyed plants × highest number of diseased levels) × 100.

### 2.7 Effects of B105-8 treatments on the activities of soil neutral phosphatase, urease, invertase, and catalase

The preparation and inoculation method of the inoculum remained identical to that detailed in section 2.5. The study included three treatments, each replicated thrice. Treatmenti (CK treatment) involved inoculating only with the pathogenic fungus; ii referred to inoculation of pathogenic fungus along with accompanying 2 mL/plant of biocontrol bacteria; iii refered to inoculation of the pathogenic fungus with 1 mL/plant of 80% carbendazim wettable powder (800 μg/mL). A 10 g of soil sample was sequentially collected each 7 days for five consecutive time periods post-second inoculation with B105-1, promptly preserved at-80°C within a refrigerator for subsequent soil enzyme activity testing utilizing soil enzyme activity kits (Nanjing Jiancheng Bioengineering Institute, Nanjing, China). These kits comprised soil urease (S-UE), neutral phosphatase (S-NP), invertase (S-SC), and catalase (S-CAT), according to the provided kit instructions for specific operation methods.

### 2.8 Preparation and identification of lipopeptide extracts from B105-8

A lipopeptide preparation was obtained from strain B105-8 employing the method delineated by Li et al. (2020b) and was preserved at 4°C until further utilization. Subsequently, the lipopeptide preparation was purified using a protein purification system employing an NGC Quest 100 Plus chromatography system. The mobile phase utilized in this process was phosphate-buffered saline (PBS). The analysis was carried out with a flow rate of 1 mL min^−1^ for 50 min and detection was conducted at a wavelength of 215 nm. Purified lipopeptides were analyzed using an UltiMate 3,000 HPLC system (Thermo Scientific Co., United States) equipped with a Supersil ODS2 column (5 μm, 4.6 mm × 150 mm) (Agilent Technologies Co., USA). The lipopeptides were dissolved in methanol to prepare 1 g L^− 1^ samples and chromatographic analysis was conducted at 215 nm with a flow rate of 1 mL min^− 1^ for 50 min. In the elution system, 0.65‰ and 0.5‰ trifluoroacetic acid (TFA) acetonitrile solutions were employed as the A-phase and B-phase, respectively.

The inhibitory activity of various fractions purified through a protein purification apparatus against *F. graminearum* was evaluated using the filter paper inhibition zone method. *F. graminearum* was inoculated in the center of the culture dish with a disk (d = 0.7 cm), and a sterile filter paper disk was placed 2 cm away from the center. A 10 μL of the purified fraction was added to the filter paper disk, while the control was supplemented with an equivalent volume of sterile water. The plates were subsequently incubated at 28°C for 3 to 5 days. The inhibitory radius was measured, the inhibition rate was calculated using the following formula and the inhibitory activity was assessed.

Inhibition rate = (diameter of inhibition zone in treatment group - diameter of inhibition zone in blank group)/diameter of inhibition zone in treatment group × 100%.

### 2.9 The effects of the optimal component from B105-8 on mycelial growth, conidial production and morphology of *Fusarium graminearum*

The antifungal components were, respectively, added to a quantitative PDA medium at concentrations of 1 μL/mL, 5 μL/mL, and 10 μL/mL. After thorough mixing, the medium was transferred to petri dishes. Subsequently, the *F. graminearum* was cultivated and sectioned into disks with a diameter of 0.7 cm, which were subsequently inoculated into the center of each petri dish. Three replications were conducted for each treatment. The control treatment contained an equivalent amount of sterile water. Following a 5-day incubation at 26°C, the diameters of the fungal colonies were measured, and the antifungal efficacies were calculated using the following formula.

A 7-mm diameter mycelial plug of *F. graminearum* was inoculated in the center of the PDA plate (9 cm in diameter). Upon growth of the colony to a diameter of 4 cm, the culture medium devoid of hyphae was removed. Subsequently, a volume of 20 mL of the optimized component derived from B105-8 was infused into the PDA plate containing the colony at concentrations of 1, 5, and 10%, with 20 mL of sterilized distilled water serving as the control. Following a soaking period of 20 min, the mycelium was carefully scraped off, leaving behind a suspension that was subsequently removed. Each treatment consisted of three replicates. Following incubation at 26°C for 72 h, 20 mL sterile water was poured into the plates to wash the spores, and the conidia concentration was recorded using a haemocytometer. The experiments were replicated twice.

A mycelial plug (7 mm in diameter) of *F. graminearum* was inoculated onto PDA plates and incubated at 26°C for 12 h. Fresh hyphae were meticulously scraped and subsequently immersed in the optimized component derived from strain B105-8 at concentrations of 1, 5, and 10%. The morphological characteristics of the hyphae were observed and compared under a light microscope.

### 2.10 Data analysis

Analysis of variance (ANOVA) was performed using SPSS Statistics 17.0 (IBM/SPSS, Armonk, NY, United States). Significant differences between treatments were assessed using Duncan’s multiple range test at a significance level of 0.05.

## 3 Results

### 3.1 Isolation, screening and re-screening of biocontrol bacteria

**Figure 1:**
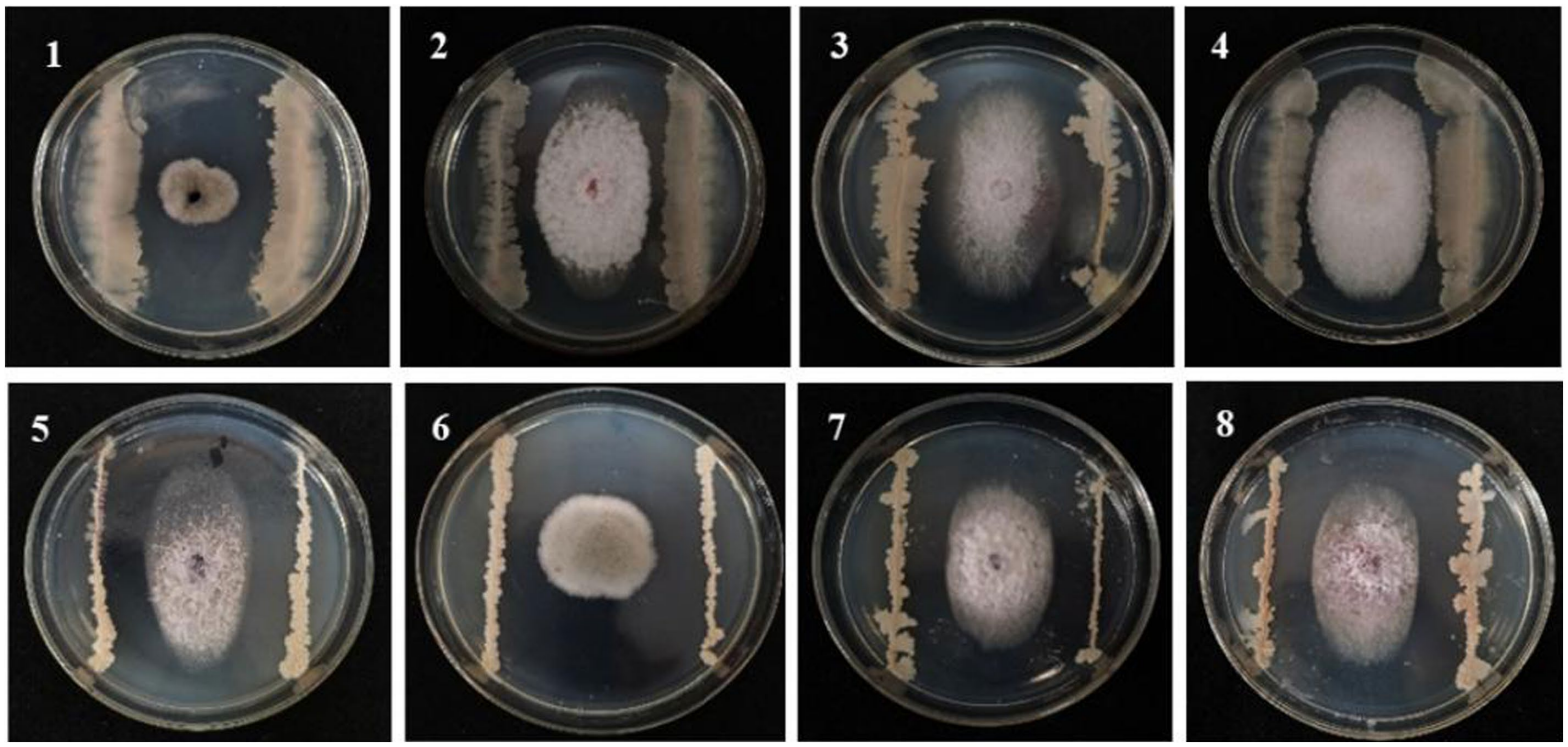
Antagonistic effect of biological bacteria B105-8 on the mycelial growth of pathogenic fungi on the sixth day after inoculation. 1, *Bipolaris zeicola*, 2, *Fusarium equiseti*; 3, *Fusarium graminearum*; 4, *Fusarium avenaceum*; 5, *Fusarium subglutinans*; 6, *Nigrospora oryzae*; 7, *Fusarium verticillioide*; 8, *Fusarium turcicum*.

A total of 120 strains isolated from the maize rhizosphere soil were evaluated for their antagonistic effects against *F. graminearum* causing MRS. Ten strains showed better antagonistic effects than other strains (Supplementary Table S1). Subsequent potting re-screening showed that strain 8 exhibited superior overall performance based on comprehensive assessments of root length, bud length, initial biomass, and disease index, which also had good biocontrol potential with an average ratio of 2.26 and an average disease index of 23.08 on MSR. Strain 8 (named B105-8) was selected for subsequent experiments. Additionally, B105-8 exhibited strong antagonistic activity against pathogenic fungi causing maize disease (Supplementary Table S2; Figure 1), including *F. graminearum*, *F. avenaceum,* and *F. subglutinans.*

### 3.2 Identification of isolate B105-8

**Figure 2:**
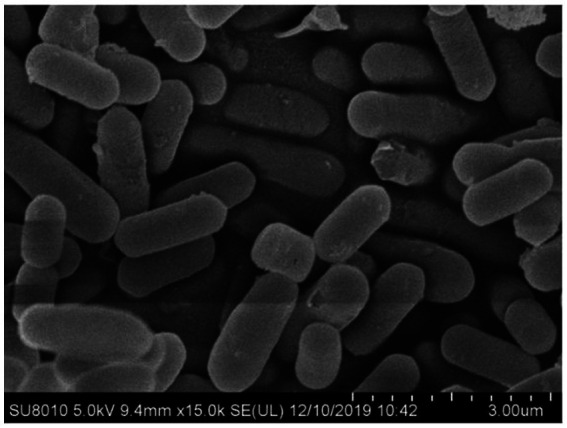
Scanning electron microscopic image of cells of *Bacillus velezensis* B105-8 cultivated at 30°C in nutrient agar broth for 24 h.

**Figure 3:**
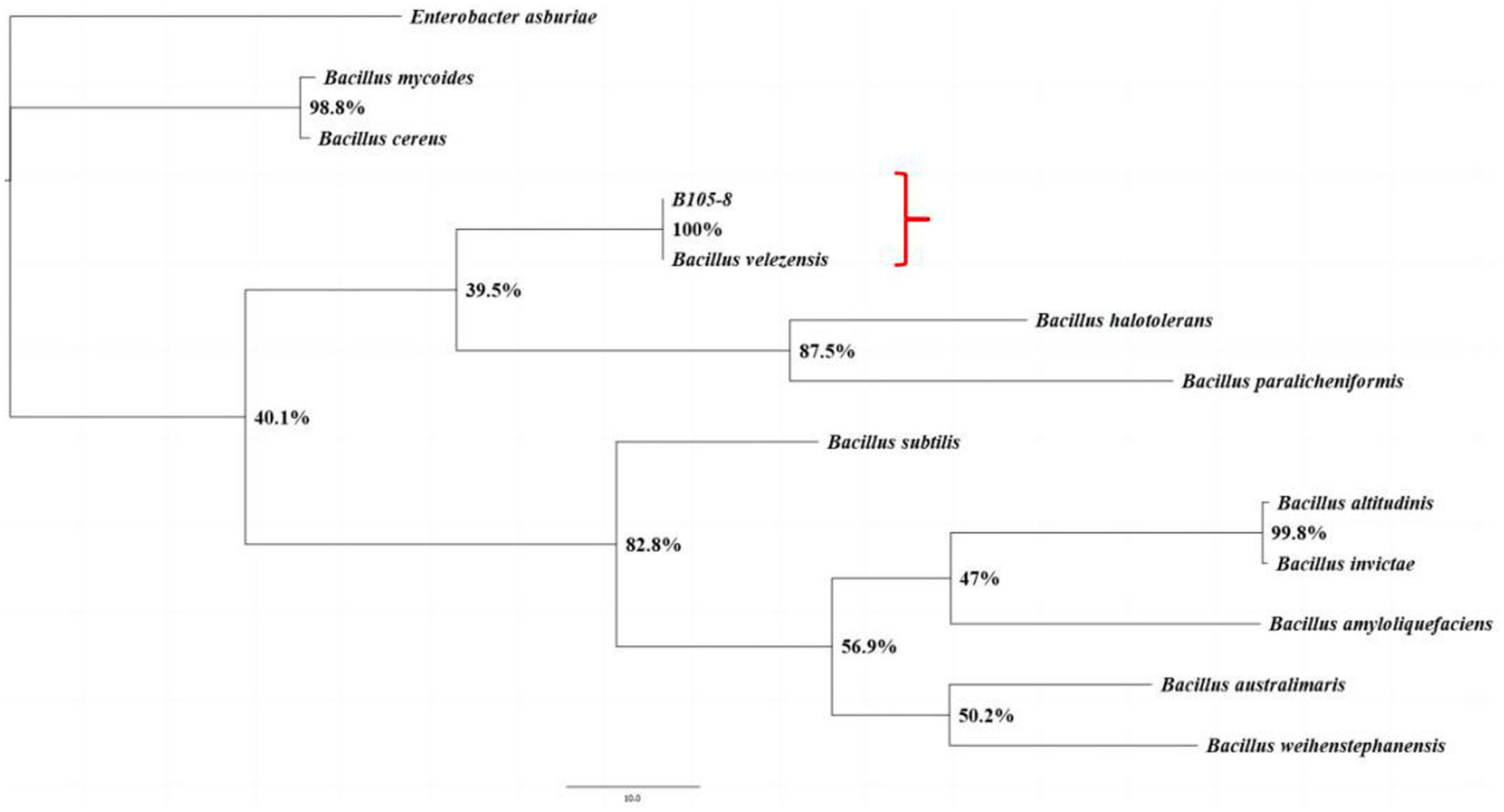
The phylogenetic analysis of the isolates B105-8 of *Bacillus velezensis* was conducted utilizing Bayesian inference considering the combined dataset of 16S ribosomal RNA and DNA gyrase subunit B. The frequency of tree sampling was 1,000 generations. Branches that received Bayesian posterior probabilities of 1.000 were set as significantly supported. *Enterobacter asburiae* was included as the outgroup.

B105-8 is a Gram-positive and rod-shaped bacterium with dimensions varying between 1.1 μm to 1.8 μm and 0.4 μm to 0.7 μm (*n* = 50), exhibiting rounded cellular ends and frequently lacking a discernible fold (Figure 2). B105-8 is an anaerobic bacteria with a distinct ability to utilize arabinose, xylose, lactose, maltose, sucrose, glucose, mannitol, mannose, sorbitol, cellobiose, fructose, and inositol, while excluding galactose, myo-inositol, and rhamnose. The bacteria also exhibited negative test results for malonate and citrate, and positive reactions to casein and catalase tests. In addition, B105-8 is capable of breaking down hydrogen peroxide, hydrolyzing gelatin, and digesting starches. The bacteria displays positive results for nitrate reduction, V-P test, and MR test, while exhibiting negative results for hydrogen sulfide test. Genomic DNA was isolated from strain B105-8, and primer sets including 1492R/27F and *gyrB-F*/*gyrB-R* were used to amplify 16S ribosomal RNA gene and DNA gyrase subunit B (*gyrB*) gene, respectively. The DNA sequences of the isolate was found to be identical. The 16S rDNA sequence and DNA gyrase subunit B of strain B105-8, as deposited in GenBank (accession No. ON059660.1 and PQ179700.1), was found to be 100% identical to that of *Bacillus velezensis* strain 34–2-1 (accession No. PP325775.1) and strain B322-1 (accession No. PP869695.1). Analysis of phylogenetic trees revealed that B105-8 and *B. velezensis* exhibited a close genetic relationship (Figure 3). Therefore, based on morphological and molecular phylogenetic analyses, strain B105-8 was finally identified as *B. velezensis*.

### 3.3 Reduction MSR following application of B105-8

**Figure 4:**
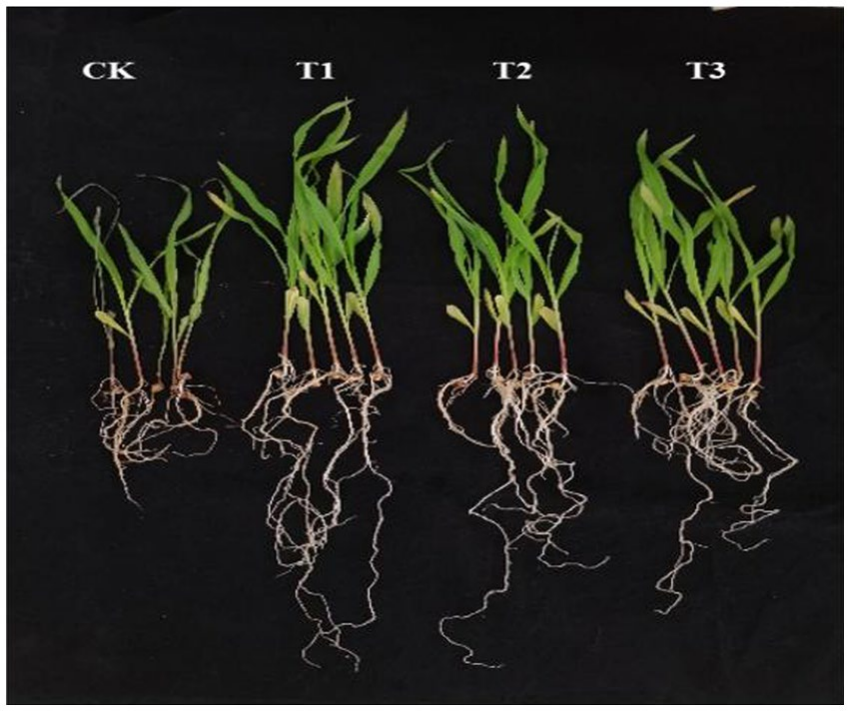
Growth status of maize root treated with the biocontrol bacterium strain B105-8 against maize stalk rot caused by *Fusarium graminearum* after 20 days in pot experiment. CK treatment involved inoculating only with *Fusarium graminearum*; T1 referred to inoculation of *Fusarium graminearum* and 1 mL/plant of B105-8; T2 represented to inoculation of *Fusarium graminearum* and 2 mL/plant of B105-8; T3 indicated inoculation of *Fusarium graminearum* and 3 mL/plant of B105-8.

As shown in Supplementary Table S3, the MSR disease index for *Fusarium graminearum* alone was close to 70%. Compared to control, B105-8 significantly reduced the MSR disease index, with control efficacy of B105-8 exceeding 60%, respectively (Supplementary Table S3). In addition, B105-8 significantly improved maize seedling growth, increased root length, plant height, and fresh weight. The optimal efficacy of total control and growth promotion was achieved with a volume of 1 mL/plant of B105-8 suspension, showing two control efficacies of 64.98 and 69.81%, respectively (Supplementary Table S3; Figure 4).

### 3.4 Effects of B105-8 treatments on the activities of soil neutral phosphatase, urease, invertase, and catalase

**Figure 5:**
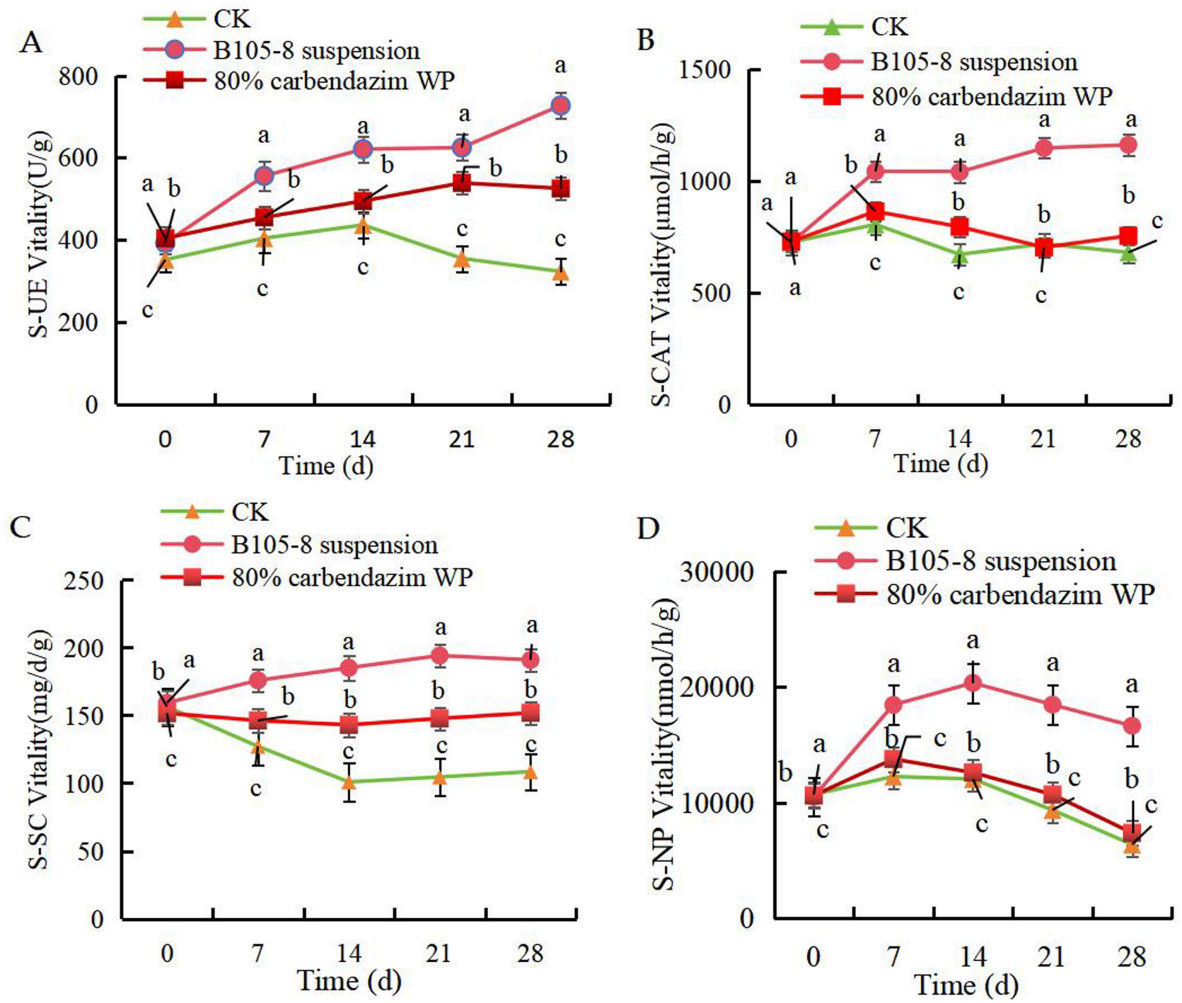
Effect of B105-8 on A, soil urease (S-UE), **B**, soil neutral phosphatase (S-NP), **C**, soil invertase (S-SC), **D**, soil catalase (S-CAT). Three treatments with 3 replicates were included: i (CK), 5 mL of conidia suspension (1 × 10° spores/mL) of Fusarium graminearum; ii (B105-8 suspension), 5 mL of conidia suspension (1 × 10° spores/mL) of *Fusarium graminearum*; iii (80% carbendazim WP), both 3 mL of conidia suspension (1 × 106 spores/mL) of *Fusarium graminearum* and 1 mL of 80% carbendazim at a concentration of 100 μg/mL.

The B105-8 suspension significantly improved the activities of S-UE (Figure 5A), S-CAT (Figure 5B) and S-SC (Figure 5C) within a 28-day post-inoculation period. However, S-NP activity showed an initial increase followed by a subsequent decline (Figure 5D), yet activity was still significantly superior to control treatment of chemical fungicide. Meanwhile, *F. graminearum* in soil significantly decreased soil activity. Overall, strain B105-8 exhibits a superior promotional effect on soil enzyme activity when compared to control treatment involving chemical fungicides. Accordingly, the application of B105-8 repaired the damage caused by *F. graminearum* on soil activity.

### 3.5 Preparation and identification of lipopeptide extracts from strain B105-8

**Figure 6:**
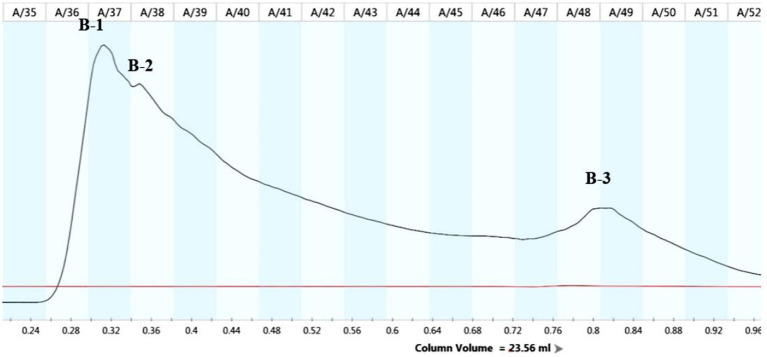
Chromatograms of lipopeptides from the biocontrol bacterium strain B105-8.

The lipopeptide extraction from strain B105-8 was analyzed by full wavelength scanning from 200 nm to 1,100 nm. The scan results revealed that the highest absorption peak of the crude lipopeptide extract occurred at a 215 nm wavelength. Therefore, the 215 nm wavelength was selected for purification and analysis by means of a protein spectrometer. Following molecular sieve elution according to the respective mass, three groups of elution peaks were obtained, each consisting of 36 to 37 vials, 38 to 40 vials, and 48 to 50 vials. These three components were sequentially named B-1, B-2, and B-3 (Figure 6).

**Figure 7:**
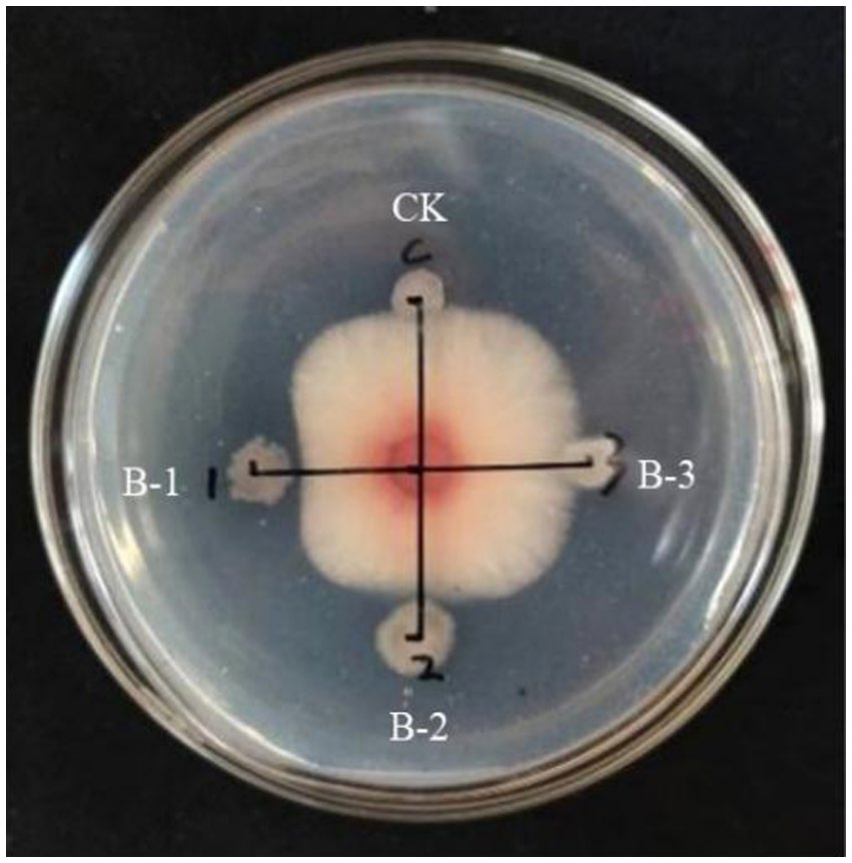
Inhibitory effect of three elution peaks of lipopeptides from the biocontrol bacterium strain. B105-8 on mycelium growth of *Fusarium graminearum*.

Determination of antifungl efficacy of the main components of B105-8 lipopeptide extract. As shown in Figure 7, it was observed that only the B-1 elution peak displayed notable antifungal activities against the mycelium, with an inhibition rate of 37.9%. The B-2 eluate peak exhibited a moderately significant impact, with an inhibition rate of 12.4%, possibly due to the presence of consequent peaks (Supplementary Table S4). In comparison, the B-3 eluate peak exhibited no inhibitory effects. Consequently, the first elution peak was retained for subsequent testing.

**Figure 8:**
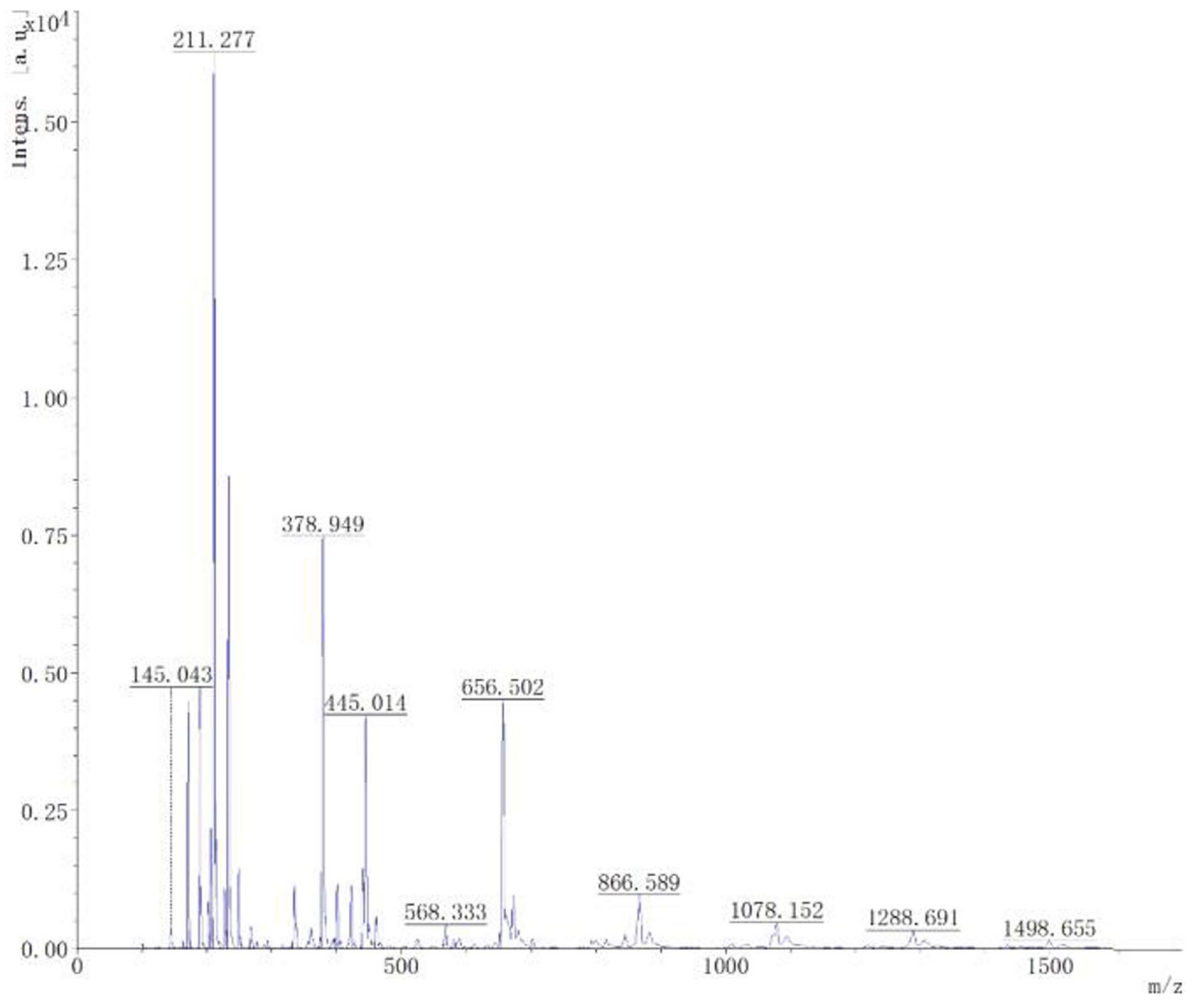
MALDI-TOF-MS analysis of the B-1 peak derived from lipopeptides from the biocontrol bacterium strain B105-8.

Mass spectrometry analysis and identification. Through matrix assisted laser desorption ionization time-of-flight mass spectrometry analysis, it could be seen from Figure 8 that the mass spectrum showed a 1,078 Da difference of 1 Da from the known C14 Bacillomycin F, C15 Iturin A, and C15 Mycosubtilin, with 1,498 Da difference of 1 Da from the known C17 fengycin A, C17 fengycin B, and C15 fengycin B. It can be inferred that the lipopeptide substance contained the compounds C14 Bacillomycin, C15 Iturin, C15 Mycosubtilin, C17, and C15 fengycin. This analysis may be attributed to the presence of double bonds in the fatty acid carbon chain, resulting in the reduction of one H atom.

**Figure 9:**
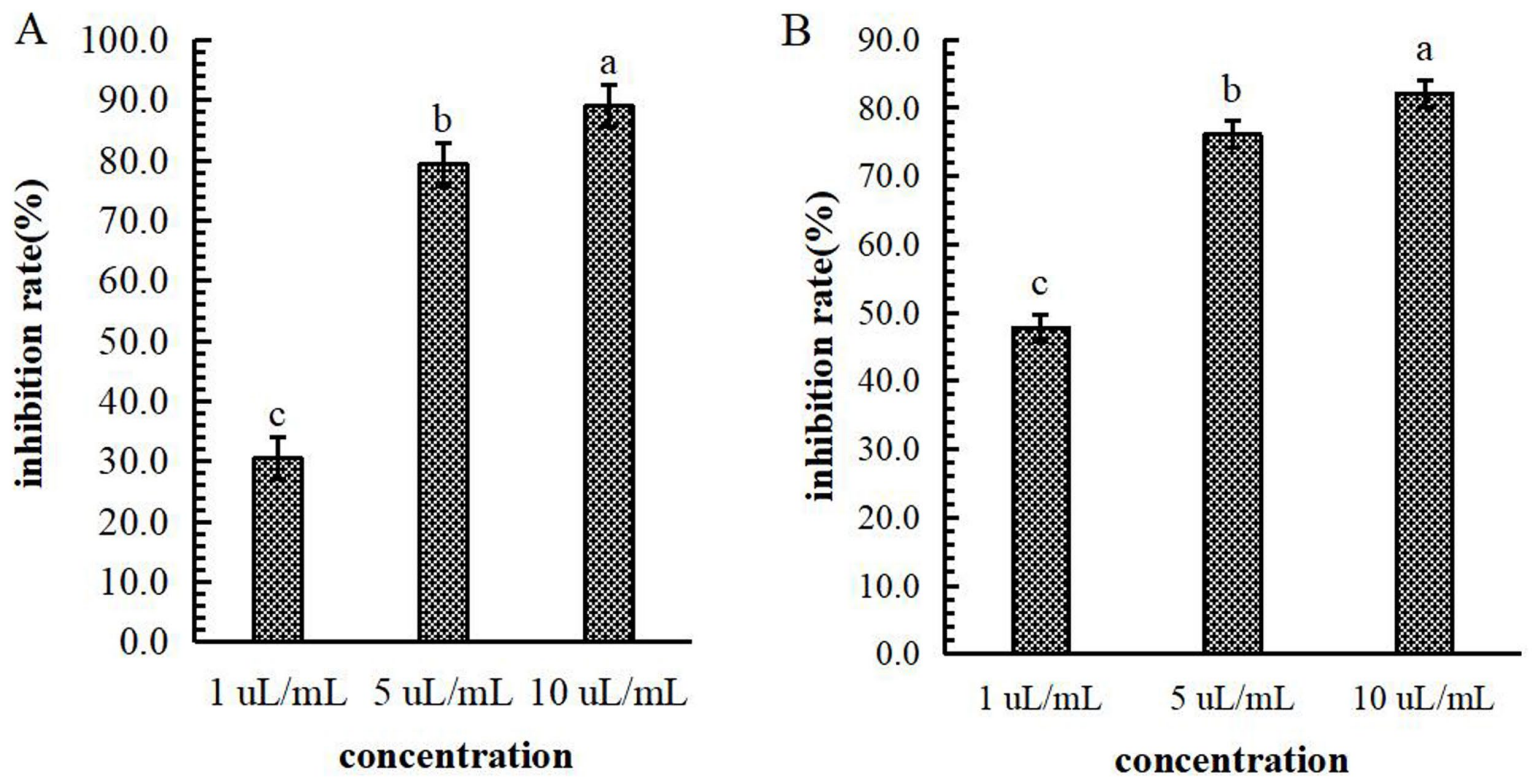
Effect of the biocontrol bacterium strain B105-8 filtrate on conidial production **(A)** and mycelial growth **(B)** of *Fusarium graminearum*. B105-8 filtrate was applied at concentrations of 1 μL/mL, 5 μL/mL, and 10 μL/mL, respectively. Error bars indicate standard errors of the means of two repeated experiments. Different letters above the bars indicate a significant difference within each group (i.e., conidial production, and mycelial growth) according to Duncan’s multiple range test (*p* < 0.05).

Mechanism of B-1 extraction from the lipopeptide extract of 105–8 against *F. graminearum.* The influence of B-1 extraction from the lipopeptide extract of 105–8 on *F. graminearum* revealed significant inhibition of conidia production and mycelial growth at concentrations of 1 μL/mL, 5 μL/mL, and 10 μL/mL, in comparison with the control (an equivalent amount of sterile water). The conidial production and mycelial growth inhibition rates at the concentration of 5 μL/mL and 10 μL/mL were significantly higher than those observed at 1 μL/mL, reaching a inhibition rate of 89.0 and 82.1% at a concentration of 10 μL/mL, respectively (Figure 9).

**Figure 10:**
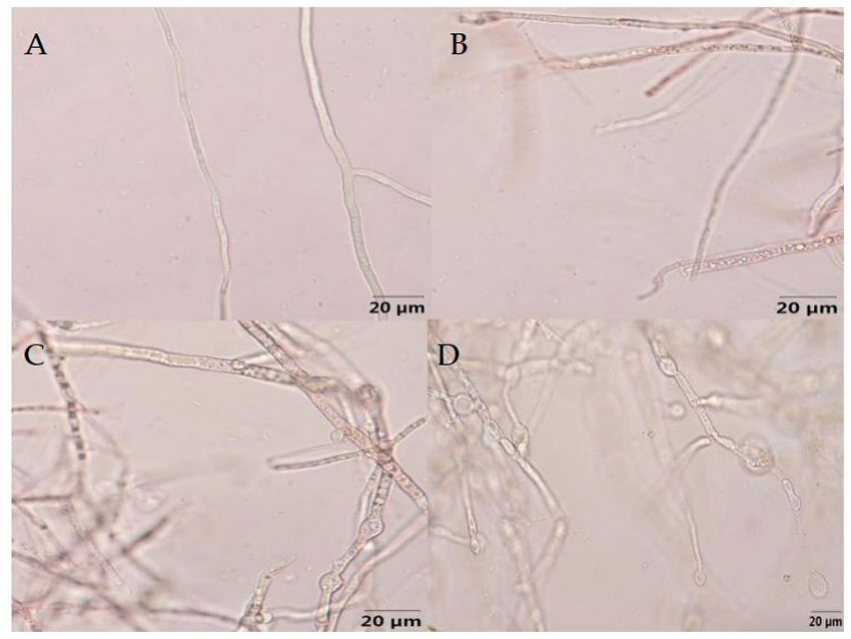
Effect of the biocontrol bacterium strain B105-8 filtrate on mycelial morphology of *Fusarium graminearum* (x 400 times). **A**, Nontreated control; **B**, **C**, and **D** indicate treatments at different concentrations of 1, 5 and 10%, respectively.

As showed in Figure 10, the mycelial growth of *F. graminearum* without the treatment of B-1 extraction exhibited normal development, smooth surface, and uniform distribution of protoplasm. Following treatment with varying concentrations of B-1 extraction, the mycelium exhibited varying degrees of uneven distribution of protoplasm and agglomerated into clumps. The upper part of the mycelium expanded and deformed, forming small vesicles, and some mycelium ruptured and broken. As the concentration increases, the number of chlamydospores increased significantly. And as the concentration increases, the various changes in the mycelium become more severe.

## 4 Discussion

Maize is one of the major crops in China, but MSR occurs nationwide and poses significant challenges to maize cultivation in China (Lu et al., 2020). *Bacillus* species have efficiently controlled various soil-borne pathogens as biocontrol control agents (Cao et al., 2021b). The discovery of *B. velezensis* originated in Spain in 2005 (Dong and Cai, 2001). Biological characterization, 16S rRNA sequencing, and phylogenetic analysis are effective methods for identifying *B. velezensis*, which was consistent with the identification methodology employed in this study. Due to these traits, the strain B105-8 was classified as *B. velezensis* in the current investigation.

Over the past two decades, emphasis has been placed on the use of beneficial advantageous microorganisms for the management of plant diseases (Kakar et al., 2018). There have been several reports on the application of biological factors to control MSR, including *Trichoderma* spp. against *F. verticillioides* causing MSR (Jambhulkar et al., 2021), *T. harzianum* against *F. verticillioides*, *F. subglutinans*, *F. moniliforme*, *F. graminearum* causing the post-flowering stalk rots, *B. cereus* against Fusarium verticillioides (Lizárraga-Sánchez et al., 2015). Biological control of *Bacillus* genus has been reported to suppress plant diseases caused by various soil-borne pathogens (Ding et al., 2024; Luo et al., 2023). Moreover, *Bacillus* spp. has become one of the most studied plant growth-promoting rhizobacteria (PGPR) in recent years due to its wide variety of sources, high natural survival rate, and inhibition of multiple plant pathogens together with environmental friendliness (Aly et al., 2022). However, there are few reports on the application of *B. velezensis* in the prevention and control of MSR. In this study, a *B. velezensis* strain was isolated from the maize rhizosphere, demonstrating powerful efficacy against MSR caused by *F. graminearum* and possessing substantial biocontrol potential and development prospects.

*B. velezensis* exhibits a broad-spectrum antimicrobial activity, effectively mitigating a range of plant diseases. Wang et al. (2023) demonstrated that *B. velezensis* 1–10, isolated from acidic soil of tea gardens, inhibited *Glomerella cingulata* and *Athelia rolfsii* causing tomato and grape diseases, offering significant potential for sustainable agriculture and fruit preservation. Yan et al. (2020) showed that *B. velezensis* had good antimicrobial activity, effectively controlling potato late blight in both greenhouse and field, and promoting potato plant growth; the field control effect was 42.43%. In addition, *B. velezensis* also showed significant antimicrobial activity against *Colletotrichum* (Huang et al., 2017), *Botrytis cinerea* (Jiang et al., 2018) and *Botryosphaeria dothide*a (Yuan et al., 2022). Therefore, *B. velezensis* has been proved to be effective in controlling a variety of fungal and bacterial diseases in fruits and vegetables, and has excellent biocontrol potential (Xu et al., 2021; Li et al., 2020b). These results further confirmed the potent and broad antagonism of *B. velezensis* against various pathogens. This conclusion is substantially consistent with the findings of this study, where B105-8 exhibited antagonistic potency against various fungi causing maize disease, including *F. graminearum*, *F. avenaceum*, and *F. subglutinans*, offering unique advantages in maize disease prevention and control.

Today, it is universally recognized that antimicrobial lipopeptides (LPs) play a major role in mitigating plant diseases (Rabbee and Baek, 2020). Among the Bacillus lipopeptides, surfactin, iturin, fengycin and bacillomycin have been the most extensively researched (Penha et al., 2020). Different strains of *Bacillus* spp. exhibit variation in their capacity to produce LPs. Some isolates currently produce two or three types of LPs, while others produce only one type (Wang et al., 2023). Yousfi et al. (2024) found that *B. velezensis* 32a produced various isoforms of the major cyclic lipopetide families, including surfactin (C12, C13, C14, and C15), fengycin (A: C14, C15, C18 or B C16 and B C15), and iturin A or mycosubtilin (C14, C15, C16, and C17). Headspace solid-phase microextraction and GC–MS detection indicated that *B. velezensis* 1–10 produced 22 volatile organic compound (VOC) components, including secreted lipopeptides, siderophores and volatile compounds (Wang et al., 2023). Owing to these advantages, lipopeptides may potentially serve as viable biocontrol agents for plant disease management (Xu et al., 2021). In the study, employing a matrix assisted laser desorption ionization time-of-flight mass analysis revealed that the lipopeptide composition of B105-8 contained the compounds C14 Bacillomycin, C15 Iturin, C15 Mycosubtilin, C17, and C15 Fengycin.

Within this subgroup of substances, the iturin family compounds exhibit broad antifungal efficacy and toxicity, providing optimal biological control agents for the management of disease progression in various crops (Fan et al., 2018; Dunlap et al., 2019; Rabbee and Baek, 2020). Surfactin A, iturin A, and fengycin B are the primary antifungal substances produced by *B. velezensis* (Rabbee and Baek, 2020). Fengycin B mainly induces structural changes in fungal spores (Liu and Sun, 2021). Surfactin A inhibits fungal mycelial growth and spore germination, while iturin A inhibits mycelial growth and induces exocytosis of fungal protoplasts, leading to spore deformities. Iturin A and surfactin A possess the ability to activate defense genes in host plants, resulting in reduced disease symptoms (Sarwar et al., 2018; Fujita and Yokota, 2019; Thimon et al., 1995; Wang et al., 2020b). The results are in line with our research findings. This research showed substantial reductions in mycelial growth, spore production, and spore germination of *F. graminearum*, favoring mycelial deformation. Thus, it is assumed that the inhibitory components of *B. velezensis* B105-8 are predominantly Fengycin B and iturin A.

Many studies have reported the biocontrol potency of *Bacillus* spp. against *Fusarium* spp. in MSR based on *in vitro* and field evaluations. There are many active enzymes with catalytic functions in soil, which can help plants remove harmful substances from themselves. S-SC can catalyze the hydrolysis of sucrose to produce glucose and fructose, and its activity can reflect the organic matter content and microbial biomass in the soil. The activity of SC is positively correlated with soil fertility and can also increase soluble substances in the soil. S-CAT decomposes hydrogen peroxide, which is detrimental to plant growth, into water and oxygen, reducing its adverse effects on plants and promoting plant growth (Bai et al., 2024). S-UE is also related to the organic matter content and microbial biomass in soil, and is not only one of the sources of plant nitrogen, but also promotes the utilization efficiency of urea nitrogen fertilizer. Singh et al. (2021) found that the incorporation of three biocontrol strains, including *B. velezensis*, significantly increased soil S-CAT activity. Wei et al. (2020) found that *B. velezensis* could enhance S-UE, S-NP, and S-SC activities. *B. velezensis* promotes plant growth and inhibits the propagation of soil-borne pathologies (Ercole et al., 2023; Cao et al., 2021b). In the present study, it was demonstrated that the B105-8 bacterial suspension noticeably enhanced the soil activity of S-UE, S-CAT and S-SC and repaired soil damage caused by *F. graminearum*. This is consistent with the results of previous reports. B105-8 effectively reduced MSR caused by *F. graminearum* with an average efficacy of 69.8%.

Subsequently, the analysis of antifungal active substances, fermentation process optimization, and field application effects of B105-8 will be further analyzed. The research results will provide theoretical support for the development of more efficient, low toxic and stable biological agents.

## 5 Conclusion

In the study, a soil-isolated strain B105-8 was identified as *B. velezensis*, demonstrated a broad-spectrum against various pathogens causing maize diseases, which effectively controlled MSR, exhibiting a high control efficacy of 67.40% and growth-promoting effect. B105-8 could effectively improve the defense enzyme systems of maize and promote the activities of soil enzyme. The antifungal compound B-1, extracted from B105-8, revealing inhibitory effects against *F. graminearum*. Analysis through mass spectrometry indicated potential presence of C14 Bacillomycin, C15 Iturin, C15 Mycosubtilin, C17, and C15 fengycin in B-1. In pot experiments, a 5 μL/mL concentration of B-1 exhibited 69% control on MSR, enhancing maize root elongation, elevation, and fresh weight. At 10 μL/mL, B-1 showed 89.0 and 82.1% inhibition on spore production and mycelial growth, causing hyphal deformities. B-1 demonstrated stability, its solid fermentation product maintained a concentration of 1.26 × 10^11^ CFU/g for 6 months at room temperature. In conclusion, *B. velezensis* B105-8 demonstrated potential as a biocontrol agent for MSR. Next, we will focus on synthesizing antifungal substances from B105-8, aiming to provide materials for the development of novel biocontrol agents against *F. graminearum* causing MSR and advancing applied research.

## Data Availability

The data that confirm the study's conclusions are freely accessible in GenBank at the National Center for Biotechnology Information, accession number ON059660.1 and PQ179700.1 (https://www.ncbi.nlm.nih.gov/genbank/).
