# Supplementary material for: Bacillus velezensis B105-8, a potential and efficient biocontrol agent in control of maize stalk rot caused by Fusarium graminearum
Source: Front Microbiol. 2024 Oct 16;15:1462992. doi: 10.3389/fmicb.2024.1462992 (PMC11522856; doi:10.3389/fmicb.2024.1462992)
Supplement: Supplementary file 1 [file Table_1.DOCX]

Supplementary Table S1**.** Antagonistic effect of ten biological bacteria strains on *Fusarium graminearum* causing maize stalk rot

| Antagonistic Strains No. | Maximum/Minimum radius^a^ | Root length (cm) | Bud length (cm) | Fresh weight(g) | Disease index^b^ |
| --- | --- | --- | --- | --- | --- |
| 1 | 2.50 | 6.98 | 5.69 | 5.22 | 27.91±0.41c |
| 2 | 1.90 | 6.89 | 4.79 | 4.62 | 31.08±0.57d |
| 3 | 1.55 | 5.68 | 4.05 | 4.49 | 31.37±0.04d |
| 4 | 2.19 | 7.16 | 5.29 | 5.11 | 27.10±0.58c |
| 5 | 2.03 | 7.81 | 5.43 | 5.02 | 27.67±0.23c |
| 6 | 2.45 | 8.44 | 5.45 | 5.31 | 25.75±0.69b |
| 7 | 1.81 | 6.55 | 3.95 | 4.72 | 27.89±0.51c |
| 8 | 2.26 | 8.69 | 5.45 | 5.89 | 23.08±0.59a |
| 9 | 1.57 | 5.18 | 3.83 | 4.31 | 33.45±0.40e |
| 10 | 1.17 | 6.09 | 4.52 | 4.67 | 29.89±0.28d |

^a^ Values in the column indicate mean of the maximum/ minimum radius of the pathogens; ^b^ Values in the column indicate Mean ± standard error (SE) of two repeated experiments. Values followed by different letters were signiﬁcantly different according to Duncan’s multiple range tests (*P* < 0.05).
