# Supplementary material for: Bacillus velezensis B105-8, a potential and efficient biocontrol agent in control of maize stalk rot caused by Fusarium graminearum
Source: Front Microbiol. 2024 Oct 16;15:1462992. doi: 10.3389/fmicb.2024.1462992 (PMC11522856; doi:10.3389/fmicb.2024.1462992)
Supplement: Supplementary file 2 [file Table_2.DOCX]

Supplementary Table S2**.** Assessment of biological bacteria B105-8 antagonistic activity against eight fungal pathogens isolated from maize in vitro

| Phytopathogen Strains No. | Mean ± SE^a^ |
| --- | --- |
| *Bipolaris zeicola* | 1.03±0.005e |
| *Fusarium equiseti* | 1.86±0.003c |
| *F. graminearum* | 2.26±0.008b |
| *F. avenaceum* | 2.05±0.005b |
| *F. subglutinans* | 2.37±0.003a |
| *Nigrospora oryzae* | 1.03±0.003e |
| *F. verticillioide* | 1.65±0.006d |
| *F. turcicum* | 1.89±0.008c |

^a^Values in the column indicate mean ± standard error (SE) of the maximum/ minimum radius of the pathogens. Values followed by different letters are significantly different according to Duncan’s multiple range tests (*P* < 0.05).
