# Supplementary material for: Bacillus velezensis B105-8, a potential and efficient biocontrol agent in control of maize stalk rot caused by Fusarium graminearum
Source: Front Microbiol. 2024 Oct 16;15:1462992. doi: 10.3389/fmicb.2024.1462992 (PMC11522856; doi:10.3389/fmicb.2024.1462992)
Supplement: Supplementary file 3 [file Table_3.DOCX]

Supplementary Table S3. Determination of the control efficacy on the biocontrol bacterium B105-8 against maize stalk rot caused by *Fusarium graminearum* in pot experiments

| Repeat times | Treatment | Fresh weight (g) | Plant height  (cm) | Length of root (cm) | Disease index | Disease reduction (%) |
| --- | --- | --- | --- | --- | --- | --- |
| 1 | （CK） | 1.54±0.01c | 30.00±1.21b | 12.46±0.09b | 65.91±0.41a | --- |
|  | T1 | 2.38±0.02a | 34.60±0.29a | 35.61±0.23a | 23.08±0.59c | 64.98 |
|  | T2 | 2.33±0.01ab | 32.28±0.68ab | 30.74±0.42a | 24.17±0.04c | 63.33 |
|  | T3 | 2.31±0.03b | 31.67±0.32b | 27.36±2.81a | 26.10±0.58b | 60.40 |
| 2 | （CK） | 1.54±0.01c | 32.9±0.46c | 13.40±0.75d | 76.67±0.23a | --- |
|  | T1 | 2.45±0.01a | 37.3±0.17a | 36.24±0.57a | 23.15±1.10b | 69.81 |
|  | T2 | 2.40±0.03ab | 35.1±0.64b | 32.91±0.64b | 23.50±0.60b | 69.35 |
|  | T3 | 2.37±0.01b | 34.6±0.78bc | 27.60±0.64c | 24.84±0.71b | 67.60 |

Note: The data is the average of three measurements. CK is only inoculated with pathogenic fungus; T1 refers to inoculation of pathogenic fungus and biocontrol bacteria 1 mL/plant; T2 refers to inoculation of pathogenic fungus and biocontrol bacteria 2 mL/plant; T3 refers to inoculation of pathogenic fungus and biocontrol bacteria 3 mL/plant. Different letters in the table indicate significant differences (*P* = 0.05), the same below.
