# Supplementary material for: Bacillus velezensis B105-8, a potential and efficient biocontrol agent in control of maize stalk rot caused by Fusarium graminearum
Source: Front Microbiol. 2024 Oct 16;15:1462992. doi: 10.3389/fmicb.2024.1462992 (PMC11522856; doi:10.3389/fmicb.2024.1462992)
Supplement: Supplementary file 4 [file Table_4.DOCX]

| Lipopeptide extract components | Mycelium diameter (cm) | Inhibition rate（%） |
| --- | --- | --- |
| B-1 | 1.45 | 37.90 a |
| B-2 | 1.78 | 12.40 b |
| B-3 | 1.91 | 4.70 c |
| CK | 2.00 | -- |

Supplementary Table S4. Inhibitory effect of three elution peaks of lipopeptides from the biocontrol bacterium strain B105-8 on mycelium growth of *Fusarium graminearum*
