# Supplementary material for: Bacillus velezensis B105-8, a potential and efficient biocontrol agent in control of maize stalk rot caused by Fusarium graminearum
Source: Front Microbiol. 2024 Oct 16;15:1462992. doi: 10.3389/fmicb.2024.1462992 (PMC11522856; doi:10.3389/fmicb.2024.1462992)
Supplement: Supplementary file 5 [file Table_5.DOCX]

Supplementary Table S5. Sequences used for concatenated alignment

| Strain | 16S rDNA | *Gyrb* *gene* |
| --- | --- | --- |
| B105-8 | ON059660.1 | PQ179700.1 |
| *Bacillus velezensis* | PP325775.1 | PP869695.1 |
| *B. subtilis* | AB325584.1 | MW401279.1 |
| *B. halotolerans* | PP829319.1 | MW879358.1 |
| *B. paralicheniformis* | KY694465.1 | MT949533.1 |
| *B. altitudinis* | PP993948.1 | OQ408194.1 |
| *B. australimaris* | JX680098.1 | JX680175.1 |
| *B. invictae* | JN699028.2 | JX183206.1 |
| *B. weihenstephanensis* | AB681419.1 | EF210262.1 |
| *B. mycoides* | OR029687.1 | AY265514.1 |
| 1. *cereus* | KC990812.1 | AY265477.1 |
| *B. amyloliquefaciens* | KX518657.1 | JX014631.1 |
| *Enterobacter asburiae* | HE589462.1 | OR146995.1 |
